# T-helper 17 cell cytokines and interferon type I: partners in crime in systemic lupus erythematosus?

**DOI:** 10.1186/ar4499

**Published:** 2014-03-06

**Authors:** Zana Brkic, Odilia BJ Corneth, Cornelia G van Helden-Meeuwsen, Radboud JEM Dolhain, Naomi I Maria, Sandra MJ Paulissen, Nadine Davelaar, Jan Piet van Hamburg, Paul L van Daele, Virgil A Dalm, P Martin van Hagen, Johanna MW Hazes, Marjan A Versnel, Erik Lubberts

**Affiliations:** 1 Department of Immunology, Erasmus MC, University Medical Center, Rotterdam, P.O. Box 1738, Rotterdam 3000 DR, The Netherlands; 2 Department of Rheumatology, Erasmus MC, University Medical Center, Rotterdam, P.O. Box 1738, Rotterdam 3000 DR, The Netherlands

## Abstract

**Introduction:**

A hallmark of systemic autoimmune diseases like systemic lupus erythematosus (SLE) is the increased expression of interferon (IFN) type I inducible genes, so-called IFN type I signature. Recently, T-helper 17 subset (Th17 cells), which produces IL-17A, IL-17F, IL-21, and IL-22, has been implicated in SLE. As CCR6 enriches for Th17 cells, we used this approach to investigate whether CCR6^+^ memory T-helper cells producing IL-17A, IL-17F, IL-21, and/or IL-22 are increased in SLE patients and whether this increase is related to the presence of IFN type I signature.

**Methods:**

In total, 25 SLE patients and 15 healthy controls (HCs) were included. SLE patients were divided into IFN type I signature-positive (IFN^+^) (*n* = 16) and negative (IFN^-^) (*n* = 9) patients, as assessed by mRNA expression of IFN-inducible genes (IFIGs) in monocytes. Expression of IL-17A, IL-17F, IL-21, and IL-22 by CD4^+^CD45RO^+^CCR6^+^ T cells (CCR6^+^ cells) was measured with flow cytometry and compared between IFN^+^, IFN^-^ patients and HCs.

**Results:**

Increased percentages of IL-17A and IL-17A/IL-17F double-producing CCR6^+^ cells were observed in IFN^+^ patients compared with IFN^-^ patients and HCs. IL-17A and IL-17F expression within CCR6^+^ cells correlated significantly with IFIG expression. In addition, we found significant correlation between B-cell activating factor of the tumor necrosis family (*BAFF*)–a factor strongly correlating with IFN type I - and IL-21 producing CCR6^+^ cells.

**Conclusions:**

We show for the first time higher percentages of IL-17A and IL-17A/IL-17F double-producing CCR6^+^ memory T-helper cells in IFN^+^ SLE patients, supporting the hypothesis that IFN type I co-acts with Th17 cytokines in SLE pathogenesis.

## Introduction

Systemic lupus erythematosus (SLE) is a debilitating systemic autoimmune disease characterized by the production of autoreactive antibodies and multiorgan inflammation [1]. A hallmark of systemic autoimmune diseases is the increased expression of interferon (IFN) type I in both blood and disease-affected tissues [2]. About half of the SLE patients exhibit an IFN type I signature or upregulation of IFN type I-induced genes (IFIGs), which have been found to correlate with disease activity and severity [3-5].

Another key factor in the pathogenesis of SLE, apart from IFN type I, is interleukin-17A (IL-17A) [6-9]. IL-17A is produced by several immune cell types, including CD4^+^ T cells (Th17 cells), CD8^+^ T cells, CD4-CD8-CD3^+^ (double-negative, DN) T cells, natural killer cells, γδ-T cells, and mast cells [10,11]. Naïve CD4^+^ T cells differentiate to Th17 cells under the influence of IL-6 and TGF-β [12]. The expansion and stability of the Th17 population is regulated by IL-21 and IL-23, respectively [13,14]. C57BL/6-lpr/lpr mice that lack IL-23 receptor signaling are protected for SLE development [15]. In SLE patients, increased plasma levels of IL-17A correlate with disease activity (SLEDAI) [6]. In addition, in peripheral blood of SLE patients, an increased number of IL-17-producing cells is observed. These cells correlate with disease activity and decrease with treatment [7,8]. IL-17-producing cells have also been found in several affected organs of SLE patients [7,9].

Co-activity between IFN type I and IL-17/Th17 cells has been suggested in autoimmune diseases [16,17]. In experimental autoimmune encephalomyelitis (EAE), a mouse model for multiple sclerosis (MS), IFN type I treatment caused exacerbation if the disease was Th17 driven but was effective if the disease was Th1 driven [17]. In the same study, MS patients that did not respond to IFN type I therapy had higher serum levels of IL-17A before therapy onset [17]. These two observations suggest additional effects of the IFN type I and Th17 system co-acting in the pathogenesis of autoimmune diseases.

Co-activity of IFN type I and Th17 pathways has also been suggested for SLE by the Ro52/TRIM21^-/-^ mouse model. Ro52/TRIM21 is involved in the ubiquitination of interferon regulatory factors (IRFs), a process that limits the IFN type I response [18]. After ear tagging, Ro52/TRIM21^-/-^ mice develop an SLE-like phenotype [19]. Interestingly, when these mice are crossed on an IL-23p19^-/-^ mouse line, they do not develop SLE, indicating that the development of an SLE phenotype through enhanced IFN type I production in these mice is dependent on the IL-17/Th17 pathway.

Yet another important factor involved in the pathogenesis of SLE is B cell-activating factor of the tumor necrosis factor family (*BAFF*). *BAFF* transgenic mice develop lupus-like disease [20], and increased expression of *BAFF* protein has been found in SLE patients, correlating with increased disease activity [21-23]. We previously described a strong correlation between *BAFF* mRNA expression in monocytes and the IFN type I signature in primary Sjögren syndrome (pSS) patients [24]. Interestingly, IL-21, a cytokine produced by Th17 cells, in combination with *BAFF*, has been reported to induce synergistically the differentiation of human memory B cells into antibody-producing plasma cells in the absence of further co-stimulation [25]. *BAFF* is known to be involved in germinal center formation [26], a process in which IL-17 is also involved [27].

The previously mentioned literature suggests an association between the pathogenic IFN type I and Th17 pathways. So far, no studies have been performed on the co-occurrence of these pathogenic pathways in SLE patients. In this study, we report for the first time a higher percentage of IL-17A and IL-17A/F producing CCR6^+^ T-memory cells in IFN type I-positive SLE patients. Moreover, *BAFF* gene expression in monocytes correlates significantly with IL-21 expression in these CCR6^+^ cells, supporting the concept of co-activity of IFN type I, Th17, and *BAFF* in the pathogenesis of SLE.

## Methods

### Patients

The 25 patients fulfilling the American College of Rheumatology revised criteria for SLE [28] were recruited at the outpatient clinic of the Immunology Department and the Rheumatology Department of the Erasmus Medical Center Rotterdam. The level of disease activity was assessed by using the SLEDAI [29]. Fifteen healthy controls (HCs), neither with autoimmune diseases nor using corticosteroids, were included. Characteristics of patients and controls are summarized in Table 1. The Medical Ethical Review Committee of the Erasmus MC approved the study, and written informed consent was obtained.

**Table 1:** Demographics and clinical characteristics of participants

**Variable**	**SLE patients**		**Healthy controls**
	**(*n*** **= 25)**		**(*n*** **= 15)**
	**IFN type I negative**	**IFN type I positive**	
	**(*n*** **= 9)**	**(*n*** **= 16)**	
Demographics			
Number of females, *n* (%)	9/9 (100%)	14/16 (88%)	15/15 (100%)
Age (years)	41.3 ± 17.5	39.8 ± 15.7	41.0 ± 14.0
Race, *n* (%)			
Caucasian	8/9 (89%)	7/16 (44%)	15/15 (100%)
Black	0/9 (0)	8/16 (50%)	0/15 (0)
Asian	0/9 (0)	0/16 (0)	0/15 (0)
Mixed race	1/9 (11%)	1/16 (6%)	0/15 (0)
Clinical			
Disease duration (years)	12.1 ± 8.0	14.4 ± 11.3	-
Mucocutaneous, *n* (%)	3/9 (33%)	10/16 (63%)	-
Arthritis, *n* (%)	7/9 (78%)	6/16 (38%)	-
Serositis, *n* (%)	1/9 (11%)	3/16 (19%)	-
Nephritis, *n* (%)	3/9 (33%)	9/16 (56%)	-
Neuropsychiatric, *n* (%)	1/9 (11%)	3/16 (19%)	-
Hematologic, n (%)	6/9 (67%)	12/16 (75%)	-
Laboratory			
ANA positivity, *n* (%)	9/9 (100%)	16/16 (100%)	-
Anti-ds-DNA positivity, *n* (%)	5/9 (56%)	10/16 (63%)	-
Treatment			
Hydroxychloroquine, *n* (%)	7/9 (78%)	11/16 (69%)	-
Corticosteroids, *n* (%)	5/9 (56%)	10/16 (63%)	-
Mycophenolate mofetil, *n* (%)	0/9 (0)	4/16 (25%)	-
Azathioprine, *n* (%)	2/9 (22%)	3/16 (19%)	-
Cyclophosphamide, *n* (%)	0/9 (0)	1/16 (6%)	-

Values are the mean ± SD, median (25% quartile,75% quartile) or number (%) of patients, depending on whether the data are continuous or dichotomous, and whether the data are normally distributed or not.

### Blood collection and isolation of monocytes

Blood was collected in clotting tubes for serum preparation (stored at -80°C) and in sodium-heparin tubes for peripheral blood mononuclear cell (PBMC) preparation, as described previously [30]. CD14-positive monocytes were isolated as described [30].

### RQ-PCR

Total RNA was isolated from purified monocytes followed by cDNA preparation and real-time quantitative polymerase chain reaction (RQ-PCR) analysis by using predesigned primer/probe sets (Applied Biosystems) [30]. For calculation of relative expression, all samples were normalized against expression of the household gene Abl [31]. Fold-change values were determined from normalized CT values by using the 2^-ΔΔCT^ method (User Bulletin; Applied Biosystems, Foster City, CA, USA).

### Flow cytometry

PBMCs were restimulated, stained, and measured with flow cytometry, as previously described [32]. For extracellular staining, CD4, CD45RO, and CCR6 monoclonal antibodies were obtained from BD Biosciences (San Diego, CA, USA), and CD25 antibodies from Biolegend Inc. (San Diego, CA, USA). For intracellular staining, IL-17A, IL-17F, IL-21, and IL-22 monoclonal antibodies were obtained from eBioscience, and IL-17A monoclonal antibodies from Biolegend Inc. Samples were measured on a FACScantoII flow cytometer (BD Biosciences). Analysis was performed by using FlowJo v7.6 research software (Tree Star Inc. Ashland, OR, USA).

### Factor analysis

The expression levels of 11 IFN type I-inducible genes (previously found to be increased in SLE) were submitted to a principal component analysis to identify correlated groups of genes to reduce data complexity. Kaiser-Meyer-Olkin measure of sampling adequacy was 0.839 with a significant Bartlett test of sphericity (*P* < 0.001). Eigenvalues were derived to assess the amount of variance explained by each component factor.

### Statistical analyses

Statistical analyses were performed by using the SPSS 20.0 package. When data were not normally distributed, values were expressed as medians with interquartile ranges (IQRs), and comparisons were made by using the nonparametric Mann–Whitney *U* test. In case of more than two samples, the nonparametric Kruskal-Wallis test was performed. Correlations were assessed by using either the Pearson correlation test for normally distributed data or Spearman rho when data were not normally distributed. Differences were considered statistically significant if *P* < 0.05.

## Results

### Prevalence of the IFN type I signature in SLE patients

**Figure 1:**
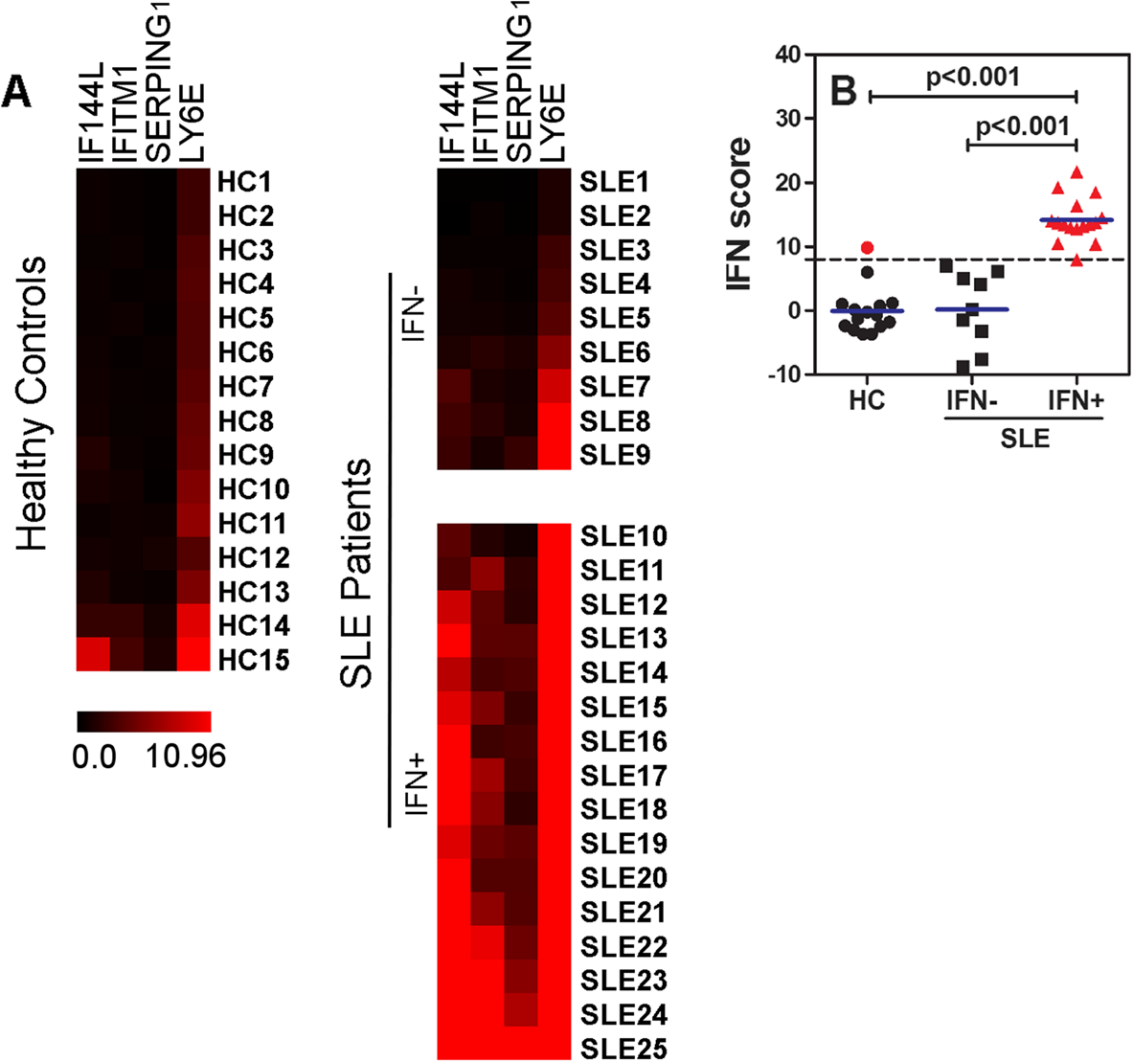
**Prevalence of IFN type I signature in SLE patients. (A)** Heatmap showing gene expression of four IFN type I-inducible genes in monocytes of SLE patients (*n* = 25) and HCs (*n* = 15). On the left, the HCs are depicted, and on the right, the SLE patients are depicted and subdivided into IFN type I signature-positive and -negative patients. Red indicates high gene expression, and cases are depicted according to IFN scores. **(B)** Distribution of IFN scores in IFN type I signature-positive and -negative patients and HCs. In red, IFN type I-positive cases are depicted. Blue lines represent medians. *P* values are shown, and to compare medians, the Mann–Whitney *U* test was used.

In monocytes of 25 SLE patients and 15 HCs, we assessed the expression levels of 11 IFIGs previously assessed in monocytes from patients with primary Sjögren syndrome (pSS) (IFI27, IFI44L, IFIT3, IFITM1, SERPING1, IFIT1, IFIT2, LY6E, IFI44, XAF1, and MXA) [24], and the expression of which was also found to be increased in SLE patients [2,3,33,34]. To reduce data complexity, expression levels of the 11 genes were submitted to a principal component analysis to identify correlated groups of genes. The results of the principal component analysis identified a subset of four genes (*IFI44L, IFITM1, SERPING1*, and *LY6E*) to explain 95% of the total variance of the 11 IFN type I-inducible genes within the SLE cohort. Given that the expression of these four IFN type I-inducible genes was not normally distributed, log transformations of expression values were performed, and IFN scores were calculated as described for pSS [24]. MeanHC and SD_HC_ of each gene in the HC-group were used to standardize expression levels. IFN scores per subject represent the sum of these standardized scores. When we set the threshold for a positive IFN type I signature at IFN score of 8 [24], 64% of SLE patients displayed an IFN type I signature, and one of the 15 HC subjects (7%) (Figure 1A,B).

### SLE patients with IFN type I signature show higher percentages of IL-17A, IL-17F, and IL-21 producing CCR6^+^ cells

**Figure 2:**
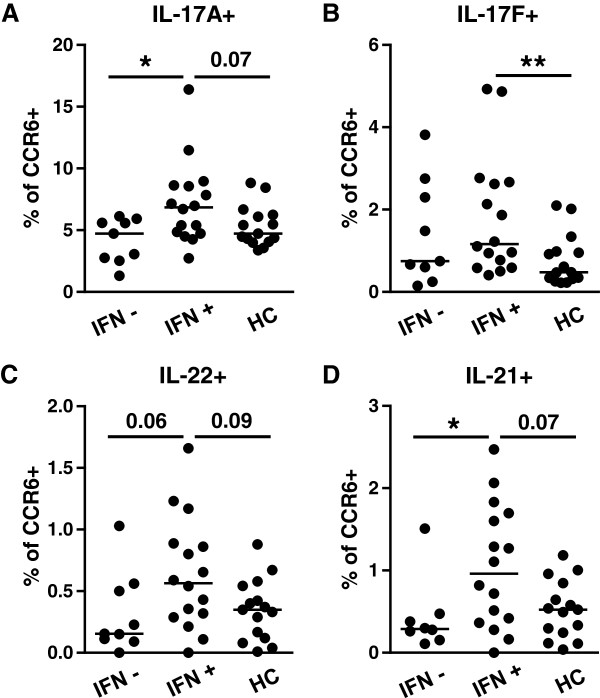
**SLE patients with IFN type I signature show higher percentages of IL-17A and IL-17A/IL-17 F producing CCR6**^**+ **^**cells. (A-D)**. Proportions of IL-17A **(A)**, IL-17 F **(B)**, IL-22 **(C)**, and IL-21 **(D)** expressing cells within CCR6^+^ memory T-cell population in PBMCs of IFN^-^ (*n* = 9) and IFN^+^ (*n* = 16) patients and HC (*n* = 15). All proportions were measured by intracellular flow cytometry. Horizontal line indicates median. *P < 0,05; **P < 0,01; to compare means, a Kruskal-Wallis test was used followed by Mann–Whitney *U* test.

Because CCR6 enriches for Th17 cells [35-37], CCR6^+^ cells were selected after gating on lymphocytes and memory Th cells (CD4^+^CD45RO^+^ cells) within PBMCs and after CD25+ cells were excluded. To investigate whether the IFN type I signature is associated with an increase in Th17 cytokines expressed by memory CCR6^+^ T cells, we measured the percentages of IL-17A, IL-17F, IL-22 and IL-21 producing CCR6^+^ T memory cells in SLE patients positive for the IFN type I signature (IFN^+^) and patients negative for the signature (IFN^-^) and HC. The percentages of CCR6^+^ cells within the CD4^+^CD45RO^+^ T-cell population and within total lymphocytes were comparable between the three studied groups (data not shown). Interestingly, the percentages of CCR6^+^IL^-^17A^+^ cells were significantly increased in IFN^+^ patients, as compared with IFN^-^ patients (*P* = 0.03), and a higher trend was observed compared with HCs (*P* = 0.07) (Figure 2A). The percentages of CCR6^+^IL^-^17F^+^ and, in particular, the IL-17A/IL-17F double producers were significantly increased in the IFN^+^ group compared with HC (*P* = 0.009) (Figure 2B, and see Additional file 1: Figure S1). The percentages of CCR6^+^IL^-2^2^+^ cells showed a higher trend for IFN^+^ versus IFN^-^ patients (^P^ = 0.06) and IFN^+^ patients versus HC (*P* = 0.09) (Figure 2C). The percentages of IL-21 expressing CCR6^+^ cells were also significantly increased in IFN^+^ patients compared with IFN^-^ patients (Figure 2D). These data suggest an association between the presence of the IFN type I signature and the expression of Th17 cytokines in SLE.

In addition, we investigated whether the Th17 cytokine production is associated with disease activity as assessed by SLEDAI scores. No significant correlations were observed between the SLEDAI scores and IL-17A and/or IL-17F expression (data not shown). Except for race, no differences in demographic, clinical, or laboratory data were found between IFN^+^, IFN^-^ SLE patients and/or HCs (Table 1).

### *BAFF* mRNA expression is correlated with IL-21 expression within CCR6^+^ memory T cells

**Figure 3:**
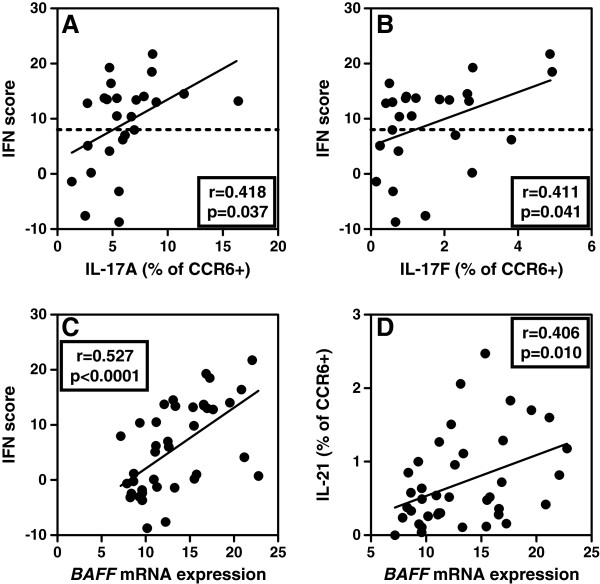
***BAFF *mRNA expression is correlated with IL-21 expression within CCR6**^**+ **^**memory T cells. (A)** Correlation between IFN score and IL-17A expression within CCR6^+^ memory T cells in SLE patients (*n* = 25). **(B)** Correlation between IFN score and IL-17 F expression within CCR6^+^ memory T cells in SLE patients (*n* = 25). **(C)** Correlation between monocyte *BAFF* mRNA expression and IFN score in SLE patients and HCs (*n* = 40). **(D)** Correlation between monocyte *BAFF* mRNA expression and IL-21 expression within CCR6^+^ cells in SLE patients and HCs (*n* = 40). The correlation coefficients (*r*) and *P* values are shown. For correlations, the Spearman rho correlation test was used in **(A)**, **(B)**, and **(D)**, and the Pearson correlation test was used in **(C)**.

Correlating the expression of IFIGs (as reflected by the total IFN score) with other parameters assessed in this study, we observed a significant positive correlation between the expression of IL-17A and IL-17F within CCR6^+^ cells and IFIG expression (Figure 3A,B). Also in this SLE cohort, IFIG expression correlated strongly with the *BAFF* mRNA expression in monocytes (*r* = 0.527; *P* < 0.0001) (Figure 3C). No correlation was observed between *BAFF* and IL-17A and/or IL-17F expression. However, we did find a significant correlation between *BAFF* mRNA and the percentages of IL-21 producing CCR6^+^ cells (*r* = 0.406; *P* = 0.010) (Figure 3D). Both *BAFF* and IL-21 are involved in the selection and activation of B cells, which is crucial in the pathogenesis of SLE, indicating that downstream factors of the IFN type I and Th17 pathways might also be associated.

## Discussion

Here we show for the first time a co-occurrence of increased IFN type I activity and increased IL-17/Th17 system in SLE patients. We found increased percentages of IL-17A, IL-17A/IL-17F, and IL-21 producing CCR6^+^ T memory cells in IFN type I-positive SLE patients. This finding further strengthens the hypothesis that IFN type I and Th17 cells, by co-acting, contribute to the pathogenesis of SLE. Further research to understand the link between these two pathways is warranted.

A possible mechanism explaining the co-occurrence of IFN type I and IL-17/Th17 immune pathway in SLE could be that both IFN type I and production of IL-6 and IL-23 by DCs are regulated through IRF-5 [38,39]. Activation of TLR signaling on DCs will then lead to simultaneous enhancement of both pathways. Evidence suggests that TLR7 activation of plasmacytoid DCs, the main producers of IFN type I, promotes and modifies Th17 cell differentiation and function [40]. IFN type I itself is also able to promote Th17 differentiation and IL-17 production through induction of STAT-3 in T cells and IL-6 in DCs [41,42]. In addition, IFN type I-conditioned monocytes differentiate into DCs, driving the development of Th17 cells from autologous naive CD4^+^ T cells [43].

In addition to the direct effect of IFN type I on Th17 cells, IFN type I may also act indirectly through the production of *BAFF*. *BAFF* is reported to be involved in DC maturation and DC-driven Th17 cell differentiation *in vitro*[44]. *BAFF* gene silencing ameliorated joint pathology and inhibited the generation of Th17 cells in the joints of a collagen-induced arthritis (CIA) mouse model [44]. In turn, IL-17A can induce the formation of neutrophil extracellular traps (NETs) [45], which could potentially provide new autoantigens to active TLRs on DCs, thereby forming a proinflammatory loop.

We find a correlation between *BAFF*, an IFN type I inducible factor, and the Th17 produced cytokine IL-21. Ettinger *et al.*[25] showed that IL-21 together with *BAFF* promotes B-cell responses by bypassing the need for T-cell help or TLR signaling. As these downstream factors are both involved in activation and selection of B cells, these findings again support the concept that IFN type I and the Th17 pathway act together in driving the disease process of SLE.

IL-21 is also produced by T follicular helper (Tfh) cells, and production by Tfh is crucial for B-cell immunity. By gating for CCR6 expression, we exclude the Tfh cells from our analysis. We therefore measured the production of IL-21 by total memory T cells (CD4^+^CD45RO^+^), which include the Tfh cells. The expression of IL-21 by these cells is significantly increased in IFN^+^ patients compared with IFN^-^ patients and HCs, and IL-21 expression by memory cells also strongly correlated with IFN score (data not shown). These data suggest that Tfh effector function may also be increased in IFN^+^ patients; however, further studies are required.

By gating on CCR6^+^ cells, we may miss certain IL-17A-producing cells, including Tfh cells. However, the percentages of IL-17A, IL-17F, and IL-22-producing cells within the CCR6^-^ population were 10– to 20-fold lower than in the CCR6^+^ population, as described previously by Acosta-Rodriguez *et al.*[35]. In addition, we did not find a difference in cytokine production between the groups when gating on total CD4^+^CD45RO^+^ memory T cells, possibly because the percentages are very small.

In contrast to others, we did not find a correlation between SLEDAI and Th17 cytokines [6]. This might be due to the relatively low patient number, which is a limitation of our study.

In addition to flow-cytometry analysis of cytokine expression by PBMCs, we also measured cytokine levels in the serum of the participants in this study (data not shown). Unfortunately, we were unable to detect IL-17A and F in most of the samples. We did find IL-22 in serum samples of all subjects, but they were not different between the groups. We also found higher levels of IL-21 in IFN^+^ patients compared with IFN^-^ patients and HCs, but we could detect IL-21 in only one third of the samples.

Although we do not show a functional link between IFN type I and the Th17 pathway, our findings provide the first support for co-occurrence of increased IFN type I activity and increased IL-17/Th17 system in SLE. The Th17-IFN type I interaction found in this study might have implications for future treatment of SLE and other systemic autoimmune diseases in which IFN type I plays a role. Preliminary results from a phase IIa trial with human IgG1κ anti-IFNα antibody, in 87 SLE patients, showed so far a 40% reduction in IFN type I-induced gene expression but no clinical effect compared with placebo (abstract; Merrill J *et al.*^*a*^). Our data indicate that IFN type I might act in concert with Th17 cytokines, paving the way for combination therapies, possibly resulting in more significant clinical effects in the future.

## Conclusion

The aim of this study was to investigate whether CCR6^+^ memory T-helper cells and their cytokine expression was increased in SLE patients. In addition, we examined whether this increase is related to the presence of IFN type I signature. In the present study, we showed that IFN^+^ patients had an increased percentage of IL-17A and IL-17A/IL-17F double-producing CCR6^+^ memory T helper cells in the blood compared to IFN negative patients and HCs. Interestingly, the IL-17A and IL-17F expression within the CCR6^+^ cells correlated significantly with IFIG expression. Moreover, monocyte *BAFF* expression in these patients correlated significantly with IL-21 producing CCR6^+^ memory T-helper cells.

Thus, this study adds new insight into the co-occurrence of the two pathogenic pathways in SLE, the IFN type I and the Th17 pathway, and showed for the first time a higher percentage of IL-17A and IL-17A/IL-17F double-producing CCR6^+^ memory T-helper cells in IFN^+^ SLE patients. These findings indicate that IFN type I co-acts with Th17 cytokines in SLE pathogenesis, and further functional studies, including understanding the mechanism, are warranted.

## Endnote

^a^J. Merrill, V. Chindalore, J. Box, N. Rothfield, J. Fiechtner, J. Sun, D. Ethgen. Results of a randomized placebo-controlled, phase 2a study of sifalimumab, an anti-interferon-alpha monoclonal antibody, administered subcutaneously in subjects with systemic lupus erythematosus [abstract]. [2011] [THU0411].

## Abbreviations

BAFF: B cell-activating factor of the tumor necrosis family; CCR6: chemokine (C-C motif) receptor 6; EAE: experimental autoimmune encephalomyelitis; HC: healthy control; IFIG: IFN-inducible gene; IFN: interferon; IL: interleukin; IRF: IFN-regulatory factor; MS: multiple sclerosis, pSS, primary Sjögren syndrome; NETs: neutrophil extracellular traps; pDC: plasmacytoid dendritic cell; SLE: systemic lupus erythematosus; SLEDAI: Systemic Lupus Erythematosus Disease Activity Index; STAT: signal transducer and activator of transcription; Tfh: T-follicular helper; Th: T helper; TLR: Toll-like receptor.

## Competing interests

The authors declare that they have no competing interests.

## Authors’ contributions

ZB and OBJC were involved in the study design and clinical and laboratory data collection, analyzed the data, and drafted and revised the manuscript. CGvH-M, NIM, SMJP, and ND were involved in laboratory data collection and revising the draft article. RJEMD, PLvD,VAD, PMvH, JMWH were involved in collection of clinical data and revising the draft. JPvH was involved in the design of the study and helped to draft the manuscript. MAV and EL were involved in study design, monitoring of data collection, and final draft and approval of the manuscript. All authors read and approved the final manuscript.

## Supplementary Material

Additional file 1: Figure S1 (A) Representative graphs of proportions of IL-17A- and IL-17F-expressing cells within CCR6^+^ memory T-cell population (defined as CD4^+^CD45RO^+^CD25-CCR6^+^) in PBMCs of IFN negative (IFN^-^) and IFN positive (IFN^+^) patients and healthy controls (HCs). (B) Representative graphs of proportions of IL-17A and IL-22-expressing cells within CCR6^+^ memory T-cell population in PBMCs of IFN^-^ and IFN^+^ patients and HCs. (C) Representative graphs of proportions of IL-21-expressing cells within CCR6^+^ memory T-cell population in PBMCs of IFN- and IFN^+^ patients and HCs. Click here for file

## References

[B1] Choi J Kim ST Craft J The pathogenesis of systemic lupus erythematosus: an update Curr Opin Immunol 2012 24 651 657 10.1016/j.coi.2012.10.004 23131610 PMC3508331

[B2] Higgs BW Liu Z White B Zhu W White WI Morehouse C Brohawn P Kiener PA Richman L Fiorentino D Greenberg SA Jallal B Yao Y Patients with systemic lupus erythematosus, myositis, rheumatoid arthritis and scleroderma share activation of a common type I interferon pathway Ann Rheum Dis 2011 70 2029 2036 10.1136/ard.2011.150326 21803750

[B3] Baechler EC Batliwalla FM Karypis G Gaffney PM Ortmann WA Espe KJ Shark KB Grande WJ Hughes KM Kapur V Gregersen PK Behrens TW Interferon-inducible gene expression signature in peripheral blood cells of patients with severe lupus Proc Natl Acad Sci U S A 2003 100 2610 2615 10.1073/pnas.0337679100 12604793 PMC151388

[B4] Bennett L Palucka AK Arce E Cantrell V Borvak J Banchereau J Pascual V Interferon and granulopoiesis signatures in systemic lupus erythematosus blood J Exp Med 2003 197 711 723 10.1084/jem.20021553 12642603 PMC2193846

[B5] Kirou KA Lee C George S Louca K Peterson MG Crow MK Activation of the interferon-alpha pathway identifies a subgroup of systemic lupus erythematosus patients with distinct serologic features and active disease Arthritis Rheum 2005 52 1491 1503 10.1002/art.21031 15880830

[B6] Chen XQ Yu YC Deng HH Sun JZ Dai Z Wu YW Yang M Plasma IL-17A is increased in new-onset SLE patients and associated with disease activity J Clin Immunol 2010 30 221 225 10.1007/s10875-009-9365-x 20107878

[B7] Yang J Chu Y Yang X Gao D Zhu L Wan L Li M Th17 and natural Treg cell population dynamics in systemic lupus erythematosus Arthritis Rheum 2009 60 1472 1483 10.1002/art.24499 19404966

[B8] Shah K Lee WW Lee SH Kim SH Kang SW Craft J Kang I Dysregulated balance of Th17 and Th1 cells in systemic lupus erythematosus Arthritis Res Ther 2010 12 R53 10.1186/ar2964 20334681 PMC2888202

[B9] Wang Y Ito S Chino Y Goto D Matsumoto I Murata H Tsutsumi A Hayashi T Uchida K Usui J Yamagata K Sumida T Laser microdissection-based analysis of cytokine balance in the kidneys of patients with lupus nephritis Clin Exp Immunol 2010 159 1 10 10.1111/j.1365-2249.2009.04031.x 19807734 PMC2802690

[B10] Hueber AJ Asquith DL Miller AM Reilly J Kerr S Leipe J Melendez AJ McInnes IB Mast cells express IL-17A in rheumatoid arthritis synovium J Immunol 2010 184 3336 3340 10.4049/jimmunol.0903566 20200272

[B11] Cua DJ Tato CM Innate IL-17-producing cells: the sentinels of the immune system Nat Rev Immunol 2010 10 479 489 10.1038/nri2800 20559326

[B12] McGeachy MJ Bak-Jensen KS Chen Y Tato CM Blumenschein W McClanahan T Cua DJ TGF-beta and IL-6 drive the production of IL-17 and IL-10 by T cells and restrain T(H)-17 cell-mediated pathology Nat Immunol 2007 8 1390 1397 10.1038/ni1539 17994024

[B13] Langrish CL Chen Y Blumenschein WM Mattson J Basham B Sedgwick JD McClanahan T Kastelein RA Cua DJ IL-23 drives a pathogenic T cell population that induces autoimmune inflammation J Exp Med 2005 201 233 240 10.1084/jem.20041257 15657292 PMC2212798

[B14] Zhou L Ivanov II Spolski R Min R Shenderov K Egawa T Levy DE Leonard WJ Littman DR IL-6 programs T(H)-17 cell differentiation by promoting sequential engagement of the IL-21 and IL-23 pathways Nat Immunol 2007 8 967 974 10.1038/ni1488 17581537

[B15] Kyttaris VC Zhang Z Kuchroo VK Oukka M Tsokos GC Cutting edge: IL-23 receptor deficiency prevents the development of lupus nephritis in C57BL/6-lpr/lpr mice J Immunol 2010 184 4605 4609 10.4049/jimmunol.0903595 20308633 PMC2926666

[B16] Ambrosi A Espinosa A Wahren-Herlenius M IL-17: a new actor in IFN-driven systemic autoimmune diseases Eur J Immunol 2012 42 2274 2284 10.1002/eji.201242653 22949326

[B17] Axtell RC de Jong BA Boniface K van der Voort LF Bhat R De Sarno P Naves R Han M Zhong F Castellanos JG Mair R Christakos A Kolkowitz I Katz L Killestein J Polman CH de Waal Malefyt R Steinman L Raman C T helper type 1 and 17 cells determine efficacy of interferon-beta in multiple sclerosis and experimental encephalomyelitis Nat Med 2010 16 406 412 10.1038/nm.2110 20348925 PMC3042885

[B18] Higgs R Ni Gabhann J Ben Larbi N Breen EP Fitzgerald KA Jefferies CA The E3 ubiquitin ligase Ro52 negatively regulates IFN-beta production post-pathogen recognition by polyubiquitin-mediated degradation of IRF3 J Immunol 2008 181 1780 1786 18641315 10.4049/jimmunol.181.3.1780 PMC2824853

[B19] Espinosa A Dardalhon V Brauner S Ambrosi A Higgs R Quintana FJ Sjostrand M Eloranta ML Ni Gabhann J Winqvist O Sundelin B Jefferies CA Rozell B Kuchroo VK Wahren-Herlenius M Loss of the lupus autoantigen Ro52/Trim21 induces tissue inflammation and systemic autoimmunity by disregulating the IL-23-Th17 pathway J Exp Med 2009 206 1661 1671 10.1084/jem.20090585 19635858 PMC2722164

[B20] Mackay F Woodcock SA Lawton P Ambrose C Baetscher M Schneider P Tschopp J Browning JL Mice transgenic for ***BAFF*** develop lymphocytic disorders along with autoimmune manifestations J Exp Med 1999 190 1697 1710 10.1084/jem.190.11.1697 10587360 PMC2195729

[B21] Becker-Merok A Nikolaisen C Nossent HC B-lymphocyte activating factor in systemic lupus erythematosus and rheumatoid arthritis in relation to autoantibody levels, disease measures and time Lupus 2006 15 570 576 10.1177/0961203306071871 17080911

[B22] Petri M Stohl W Chatham W McCune WJ Chevrier M Ryel J Recta V Zhong J Freimuth W Association of plasma B lymphocyte stimulator levels and disease activity in systemic lupus erythematosus Arthritis Rheum 2008 58 2453 2459 10.1002/art.23678 18668552

[B23] Stohl W Metyas S Tan SM Cheema GS Oamar B Xu D Roschke V Wu Y Baker KP Hilbert DM B lymphocyte stimulator overexpression in patients with systemic lupus erythematosus: longitudinal observations Arthritis Rheum 2003 48 3475 3486 10.1002/art.11354 14673998

[B24] Brkic Z Maria NI van Helden-Meeuwsen CG van de Merwe JP van Daele PL Dalm VA Wildenberg ME Beumer W Drexhage HA Versnel MA Prevalence of interferon type I signature in CD14 monocytes of patients with Sjogren’s syndrome and association with disease activity and ***BAFF*** gene expression Ann Rheum Dis 2013 72 728 735 10.1136/annrheumdis-2012-201381 22736090 PMC3618683

[B25] Ettinger R Sims GP Robbins R Withers D Fischer RT Grammer AC Kuchen S Lipsky PE IL-21 and ***BAFF***/BLyS synergize in stimulating plasma cell differentiation from a unique population of human splenic memory B cells J Immunol 2007 178 2872 2882 17312131 10.4049/jimmunol.178.5.2872

[B26] Zotos D Coquet JM Zhang Y Light A D’Costa K Kallies A Corcoran LM Godfrey DI Toellner KM Smyth MJ Nutt SL Tarlinton DM IL-21 regulates germinal center B cell differentiation and proliferation through a B cell-intrinsic mechanism J Exp Med 2010 207 365 378 10.1084/jem.20091777 20142430 PMC2822601

[B27] Hsu HC Yang P Wang J Wu Q Myers R Chen J Yi J Guentert T Tousson A Stanus AL Le TV Lorenz RG Xu H Kolls JK Carter RH Chaplin DD Williams RW Mountz JD Interleukin 17-producing T helper cells and interleukin 17 orchestrate autoreactive germinal center development in autoimmune BXD2 mice Nat Immunol 2008 9 166 175 10.1038/ni1552 18157131

[B28] Hochberg MC Updating the American College of Rheumatology revised criteria for the classification of systemic lupus erythematosus Arthritis Rheum 1997 40 1725 9324032 10.1002/art.1780400928

[B29] Bombardier C Gladman DD Urowitz MB Caron D Chang CH Derivation of the SLEDAI: a disease activity index for lupus patients: The Committee on Prognosis Studies in SLE Arthritis Rheum 1992 35 630 640 10.1002/art.1780350606 1599520

[B30] Drexhage RC van der Heul-Nieuwenhuijsen L Padmos RC van Beveren N Cohen D Versnel MA Nolen WA Drexhage HA Inflammatory gene expression in monocytes of patients with schizophrenia: overlap and difference with bipolar disorder: a study in naturalistically treated patients Int J Neuropsychopharmacol 2010 13 1369 1381 10.1017/S1461145710000799 20633309

[B31] Beillard E Pallisgaard N van der Velden VH Bi W Dee R van der Schoot E Delabesse E Macintyre E Gottardi E Saglio G Watzinger F Lion T van Dongen JJ Hokland P Gabert J Evaluation of candidate control genes for diagnosis and residual disease detection in leukemic patients using ‘real-time’ quantitative reverse-transcriptase polymerase chain reaction (RQ-PCR): a Europe against cancer program Leukemia 2003 17 2474 2486 10.1038/sj.leu.2403136 14562124

[B32] van Hamburg JP Asmawidjaja PS Davelaar N Mus AM Colin EM Hazes JM Dolhain RJ Lubberts E Th17 cells, but not Th1 cells, from patients with early rheumatoid arthritis are potent inducers of matrix metalloproteinases and proinflammatory cytokines upon synovial fibroblast interaction, including autocrine interleukin-17A production Arthritis Rheum 2011 63 73 83 10.1002/art.30093 20954258

[B33] Nikpour M Dempsey AA Urowitz MB Gladman DD Barnes DA Association of a gene expression profile from whole blood with disease activity in systemic lupus erythaematosus Ann Rheum Dis 2008 67 1069 1075 10.1136/ard.2007.074765 18063674

[B34] Lood C Amisten S Gullstrand B Jonsen A Allhorn M Truedsson L Sturfelt G Erlinge D Bengtsson AA Platelet transcriptional profile and protein expression in patients with systemic lupus erythematosus: up-regulation of the type I interferon system is strongly associated with vascular disease Blood 2010 116 1951 1957 10.1182/blood-2010-03-274605 20538795

[B35] Acosta-Rodriguez EV Rivino L Geginat J Jarrossay D Gattorno M Lanzavecchia A Sallusto F Napolitani G Surface phenotype and antigenic specificity of human interleukin 17-producing T helper memory cells Nat Immunol 2007 8 639 646 10.1038/ni1467 17486092

[B36] Annunziato F Cosmi L Liotta F Maggi E Romagnani S The phenotype of human Th17 cells and their precursors, the cytokines that mediate their differentiation and the role of Th17 cells in inflammation Int Immunol 2008 20 1361 1368 10.1093/intimm/dxn106 18820263

[B37] Ivanov II McKenzie BS Zhou L Tadokoro CE Lepelley A Lafaille JJ Cua DJ Littman DR The orphan nuclear receptor RORgammat directs the differentiation program of proinflammatory IL-17+ T helper cells Cell 2006 126 1121 1133 10.1016/j.cell.2006.07.035 16990136

[B38] Takaoka A Yanai H Kondo S Duncan G Negishi H Mizutani T Kano S Honda K Ohba Y Mak TW Taniguchi T Integral role of IRF-5 in the gene induction programme activated by Toll-like receptors Nature 2005 434 243 249 10.1038/nature03308 15665823

[B39] Lazear HM Lancaster A Wilkins C Suthar MS Huang A Vick SC Clepper L Thackray L Brassil MM Virgin HW Nikolich-Zugich J Moses AV Gale M Jr Früh K Diamond MS IRF-3, IRF-5, and IRF-7 coordinately regulate the type I IFN response in myeloid dendritic cells downstream of MAVS signaling PLoS Pathog 2013 9 e1003118 10.1371/journal.ppat.1003118 23300459 PMC3536698

[B40] Yu CF Peng WM Oldenburg J Hoch J Bieber T Limmer A Hartmann G Barchet W Eis-Hubinger AM Novak N Human plasmacytoid dendritic cells support Th17 cell effector function in response to TLR7 ligation J Immunol 2010 184 1159 1167 10.4049/jimmunol.0901706 20026744

[B41] Matikainen S Sareneva T Ronni T Lehtonen A Koskinen PJ Julkunen I Interferon-alpha activates multiple STAT proteins and upregulates proliferation-associated IL-2Ralpha, c-myc, and pim-1 genes in human T cells Blood 1999 93 1980 1991 10068671

[B42] Yasuda K Richez C Maciaszek JW Agrawal N Akira S Marshak-Rothstein A Rifkin IR Murine dendritic cell type I IFN production induced by human IgG-RNA immune complexes is IFN regulatory factor (IRF)5 and IRF7 dependent and is required for IL-6 production J Immunol 2007 178 6876 6885 17513736 10.4049/jimmunol.178.11.6876

[B43] Santini SM Lapenta C Donati S Spadaro F Belardelli F Ferrantini M Interferon-alpha-conditioned human monocytes combine a Th1-orienting attitude with the induction of autologous Th17 responses: role of IL-23 and IL-12 PLoS One 2011 6 e17364 10.1371/journal.pone.0017364 21387004 PMC3046151

[B44] Lai Kwan Lam Q King Hung Ko O Zheng BJ Lu L Local ***BAFF*** gene silencing suppresses Th17-cell generation and ameliorates autoimmune arthritis Proc Natl Acad Sci U S A 2008 105 14993 4998 10.1073/pnas.0806044105 18820032 PMC2567481

[B45] Khandpur R Carmona-Rivera C Vivekanandan-Giri A Gizinski A Yalavarthi S Knight JS Friday S Li S Patel RM Subramanian V Thompson P Chen P Fox DA Pennathur S Kaplan MJ NETs are a source of citrullinated autoantigens and stimulate inflammatory responses in rheumatoid arthritis Sci Transl Med 2013 5 178ra140 10.1126/scitranslmed.3005580 PMC3727661 23536012

