# Supplementary figures and images for: T-helper 17 cell cytokines and interferon type I: partners in crime in systemic lupus erythematosus?
Source: Arthritis Res Ther. 2014 Mar 6;16(2):R62. doi: 10.1186/ar4499 (PMC4060204; doi:10.1186/ar4499)

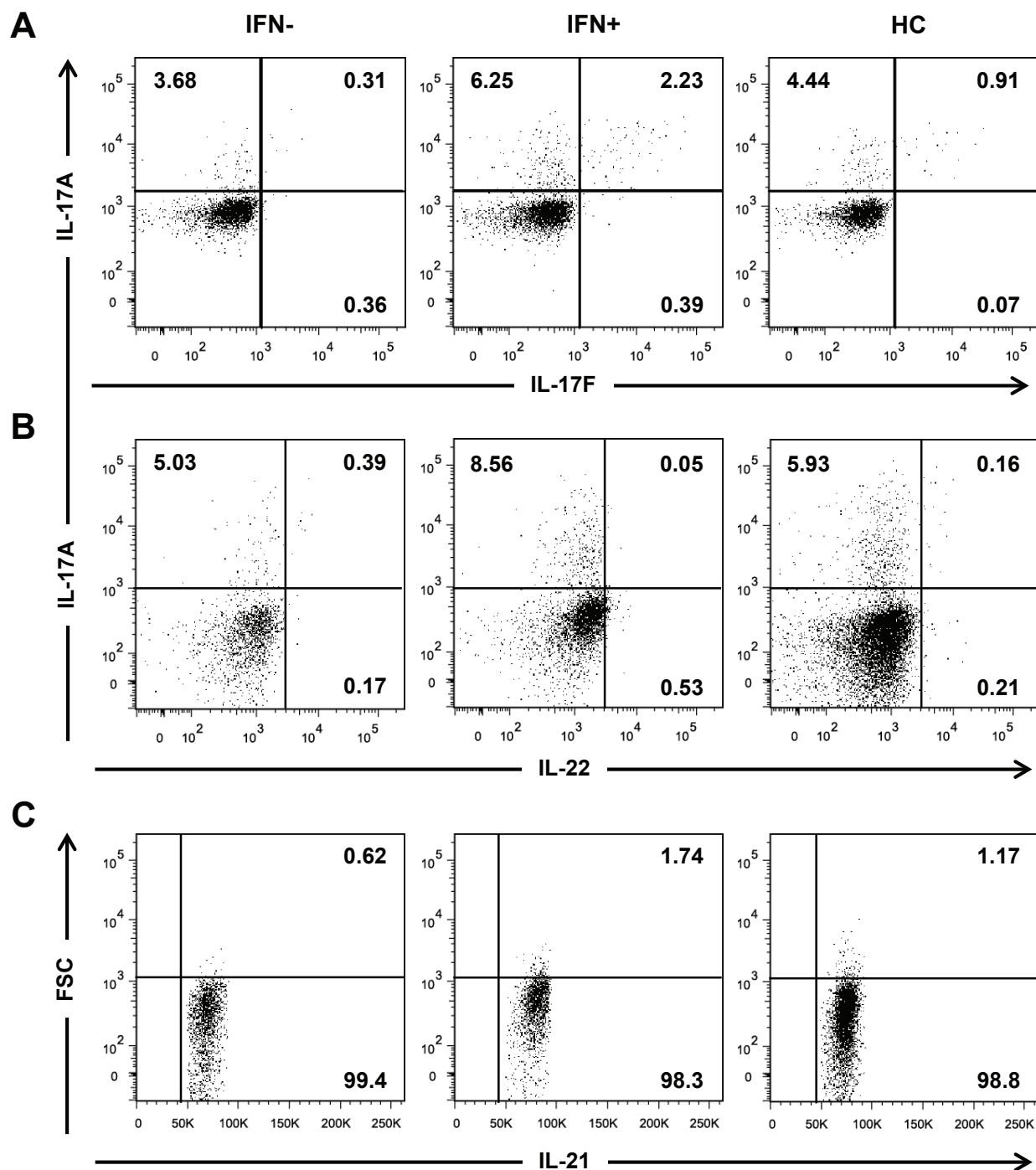

Supplement: Additional file 1: Figure S1 — (A) Representative graphs of proportions of IL-17A- and IL-17F-expressing cells within CCR6+ memory T-cell population (defined as CD4+CD45RO+CD25-CCR6+) in PBMCs of IFN negative (IFN-) and IFN positive (IFN+) patients and healthy controls (HCs). (B) Representative graphs of proportions of IL-17A and IL-22-expressing cells within CCR6+ memory T-cell population in PBMCs of IFN- and IFN+ patients and HCs. (C) Representative graphs of proportions of IL-21-expressing cells within CCR6+ memory T-cell population in PBMCs of IFN- and IFN+ patients and HCs. [file ar4499-S1.pdf]
